# Behavioral Activation for Comorbid Depression in People With Noncommunicable Disease in India: Protocol for a Randomized Controlled Feasibility Trial

**DOI:** 10.2196/41127

**Published:** 2023-11-16

**Authors:** Rayeesa Zainab, Arun Kandasamy, Naseer Ahmad Bhat, Chrishma Violla Dsouza, Hannah Jennings, Cath Jackson, Papiya Mazumdar, Catherine Elizabeth Hewitt, David Ekers, Gitanjali Narayanan, Girish N Rao, Karen Coales, Krishna Prasad Muliyala, Santosh K Chaturvedi, Pratima Murthy, Najma Siddiqi

**Affiliations:** 1 National Institute of Mental Health and Neuro Sciences Bangalore India; 2 Jindal School of Psychology and Counselling O P Jindal Global University Sonipat India; 3 Department of Health Sciences University of York & Hull York Medical School Heslington United Kingdom; 4 Valid Research LTD West Yorkshire United Kingdom; 5 School of Politics and International Studies Faculty of Social Science University of Leeds Leeds United Kingdom; 6 Department of Health Sciences University of York Heslington United Kingdom; 7 Tees Esk and Wear Valleys NHS Foundation Trust North Yorkshire United Kingdom; 8 Department of Medicine, Pharmacy, and Health Durham University North Yorkshire United Kingdom; 9 Leicestershire Partnership NHS Trust Leicester United Kingdom

**Keywords:** adaption, behavioral activation, cancer, cardiovascular disease, cultural, culture, depression, diabetes, feasibility, India, language, linguistic, mental disorder, mental health care, mental health, non-communicable disease, Patient Health Questionnaire, PHQ, randomized controlled feasibility trial, respiratory, retention, social, stroke, therapy, treatment

## Abstract

**Background:**

The increasing burden of depression and noncommunicable disease (NCD) is a global challenge, especially in low- and middle-income countries, considering the resource constraints and lack of trained human resources in these settings. Effective treatment of depression in people with NCDs has the potential to enhance both the mental and physical well-being of this population. It will also result in the effective use of the available health care resources. Brief psychological therapies, such as behavioral activation (BA), are effective for the treatment of depression. BA has not been adapted in the community health care services of India, and the feasibility of using BA as an intervention for depression in NCD and its effectiveness in these settings have not been systematically evaluated.

**Objective:**

Our objective is to adapt BA for the Indian NCD context and test the acceptability, feasibility, and implementation of the adapted BA intervention (BEACON intervention package [BIP]). Additionally, we aim to test the feasibility of a randomized controlled trial evaluation of BIP for the treatment of depression compared with enhanced usual care.

**Methods:**

Following well-established frameworks for intervention adaptation, we first adapted BA (to fit the linguistic, cultural, and resource context) for delivery in India. The intervention was also adapted for potential remote delivery by telephone. In a randomized controlled trial, we will be testing the acceptability, feasibility, and implementation of the adapted BA intervention (BIP). We shall also test if a randomized controlled feasibility trial can be delivered effectively and estimate important parameters (eg, recruitment and retention rates and completeness of follow-up) needed to design a future definitive trial.

**Results:**

Following the receipt of approval from all the relevant agencies, the development of the BIP was started on November 28, 2020, and completed on August 18, 2021, and the quantitative data collection was started on August 23, 2021, and completed on December 10, 2021. Process evaluation (qualitative data) collection is ongoing. Both the qualitative and quantitative data analyses are ongoing.

**Conclusions:**

This study may offer insights that could help in closing the gap in the treatment of common mental illness, particularly in nations with limited resources, infrastructure, and systems such as India. To close this gap, BEACON tries to provide BA for depression in NCDs through qualified NCD (BA) counselors integrated within the state-run NCD clinics. The results of this study may aid in understanding whether BA as an intervention is acceptable for the population and how feasible it will be to deliver such interventions for depression in NCD in South Asian countries such as India. The BIP may also be used in the future by Indian community clinics as a brief intervention program.

**Trial Registration:**

Clinical Trials Registry of India CTRI/2020/05/025048; https://tinyurl.com/mpt33jv5

**International Registered Report Identifier (IRRID):**

DERR1-10.2196/41127

## Introduction

Noncommunicable diseases (NCDs), also known as chronic diseases, tend to be of long duration and are the result of a combination of genetic, physiological, environmental, and behavioral factors. NCDs are the leading cause of mortality in the world, much of which is premature and avoidable [1,2]. The largest population-based study conducted in India on the prevalence of depression found that 15.1% of urban South Indians were depressed. The most frequent symptom was a depressed mood (30.8%), followed by fatigue (30%), whereas less frequently occurring symptoms included suicidal thoughts (12.4%) and speech and motor retardation (12.4%) [3]. Another study conducted in South India shows patients with ischemic heart disease and hypertension were more likely to exhibit somatization and depression symptoms, whereas patients with diabetes mellitus, those with hypertension, and those with ischemic heart disease were more likely to exhibit anxiety symptoms [4]. According to a study on comorbid depression in NCDs among older patients conducted in Punjab, India, 58.1% of study participants had depression, of which 34.1% had severe depression. Generalized anxiety disorder (GAD) was found to be present in 38.7% of cases, with 19.7% falling into the severe category. In 37.8%, GAD and depression were both identified [5]. There is increasing evidence that comorbid mental health conditions, such as depression, can greatly affect people with physical health conditions [6]. For example, people with NCDs are 2-3 times more likely to experience depression [2]. As the population grows in number and becomes older, more people with physical and mental illnesses are living longer. These persistent disorders are closely related. Several key modifiable risk factors for NCD, such as a poor diet, inactivity, smoking, and hazardous alcohol use, are made worse by poor mental health. A person is more likely to have one or more chronic illnesses if they have a mental illness, which is a risk factor for NCD [3].

The presence of depression worsens NCD symptoms, adversely impacts the quality of life, and increases the financial burden for patients and their families [2]. Therefore, it is important to recognize and treat depression to improve the overall management of NCDs and improve health, quality of life, and economic outcomes for these patients.

There is an identified need to integrate mental health care in NCD care settings to help address the high burden of comorbid depression with NCDs. However, there is a notable gap worldwide between the number of people in need of mental health care and those who receive treatment. This “treatment gap” is especially high in low- and middle-income countries (LMICs) [7,8]. It is recognized that depression management is not standardized across services and is often not available at all.

Another reason for this treatment gap would be that in developing nations, stigma is a significant barrier to people seeking care for mental health issues. Those who are stigmatized may expect to be treated unfairly and with contempt by others, which may encourage them to use unhealthy coping techniques such as withdrawal or secrecy. This tendency has been noted in research conducted in LMICs, such as Ethiopia and India. One option would be to incorporate areas of mental health treatment that influence the development of stigma, such as successful task-shifting programs, peer and health worker-delivered therapy, and case management [9]. Task shifting is a strategy that has been shown to be successful in helping to address this shortage of specialists, whereby nonspecialist health workers are trained to deliver interventions that would usually be provided by specialists [10]. This approach is supported by the World Health Organization’s (WHO’s) Mental Health Gap Action Program (mhGAP) [11].

Behavioral activation (BA) is an evidence-based, simple, structured psychotherapy for the treatment of depression, initially developed in the 1970s. BA aims to break the negative cycle of aversive behaviors and low mood by increasing engagement in activities that are associated with positive reinforcement. BA therapy is delivered by a trained practitioner, usually over a series of face-to-face therapy sessions, with “homework” tasks that are agreed upon with the person with depression to be undertaken between sessions. BA can be delivered through a number of sessions over digital and telephone platforms as well as in person [2,12,13].

Although there is interest in using BA for depression in people with NCDs, evidence for the effectiveness of this approach has not been clearly established. A systematic review conducted in 2011 found only 8 randomized controlled trials of BA in long-term conditions (with study sample sizes ranging from 20 to 105) [14]. A total of 3 trials targeted stroke; another 2 were in dementia and 1 in breast cancer. The remaining 2 included studies targeting older residents in nursing homes but did not specify the comorbid physical conditions. Since the publication of that review, the “Integrating Depression and Diabetes Treatment” (INDEPENDENT) study, a randomized controlled trial of BA delivered through a multicomponent care model in India, has shown effectiveness and cost-effectiveness for the treatment of depression in people with diabetes. However, the study intervention was complex and multifaceted, combining collaborative care, decision support, and population health management. It remains uncertain whether a briefer intervention like BA is effective for the treatment of depression in NCDs in LMICs [15].

This study aims to address this evidence gap by (1) adapting BA for the Indian NCD context; (2) testing the acceptability, feasibility, and implementation of the adapted BA intervention (BEACON intervention package [BIP]); and (3) testing the feasibility of a randomized controlled trial evaluation of BIP for the treatment of depression compared with an enhanced usual care (EUC) package.

## Methods

### Intervention Adaptation

The purpose of this step was to ensure the BA intervention content and delivery were appropriate for the Indian NCD’s cultural, linguistic, and resource context. The initial content was based on treatment manuals and workbooks from previous adaptations of BA for multimorbidity and delivery by lay counselors in the United Kingdom [16] and India [17]. The research team also looked at training packages from the WHO mhGAP [11] relevant to the delivery of BA and formative research exploring the implementation of BA in similar settings in South Asia [18].

Following adaptation frameworks, including cultural adaptation [19,20], and taking this information into account and in discussion with experts within the National Institute of Mental Health and Neuro Sciences (NIMHANS) and the wider BEACON team, the materials, plans for delivery, and training package were adapted. Revisions to the package were made through expert panel reviews, co-design workshops with NCD counselors, and feedback sessions from NCD patients and caregivers. Major revisions included modification of the workbook as a flipbook with less text and more pictures, simplification of the BA counselor’s manual, and preparation of a separate session log instead of homework tasks in between session follow-ups. The training package for the BA counselors was modified in collaboration with a BA expert from the United Kingdom.

The finalized BIP comprised a BA counselor’s manual, a session log, and a flipbook for the patient participants. The intervention could be delivered remotely by telephone over 6 sessions lasting approximately 30-40 minutes each. The training of the BA counselors focused on developing skills in communication, establishing therapeutic relationships, and using the manual and materials to deliver therapy sessions. The task of adapting BA for the Indian NCD context has already been completed.

### Feasibility Trial

#### Objectives

The objectives of the feasibility trial are to (1) test the acceptability, feasibility, and implementation of the adapted BA intervention (BIP) and (2) test if a randomized controlled feasibility trial can be delivered effectively and estimate important parameters needed to design a future definitive trial.

This will be achieved by answering the following research questions (RQs): (1) what are the recruitment and retention rates for participants in the trial? (2) What is the feasibility and acceptability of the proposed randomization and data collection procedures? (3) To what degree can the BIP be implemented as planned? (4) What is the acceptability of the BIP from the perspective of study participants and BA counselors?

#### Design

A randomized controlled feasibility trial with the nested process and economic evaluations was conducted over 12 months. The Gantt chart can be found in Multimedia Appendix 1.

The 2 trial arms are the intervention arm (BIP) and the control arm (EUC).

#### Setting

In 2010, the Ministry of Health and Family Welfare, Government of India, launched the National Programme for Prevention and Control of Cancer, Diabetes, Cardiovascular Diseases, and Stroke (NPCDCS). This includes the prevention and management of NCDs at district and subdistrict-level facilities. These facilities provide services for screening, treatment, or referral of patients with NCD but do not, at present, include the integrated detection and management of mental disorders. They do not yet cover all districts in the country [21].

The setting for the BEACON study is a district in Kolar, Karnataka, South India, where it was possible to explore the integration of mental and physical health care based on several pragmatic considerations, including the availability of NCD services that are part of the government health system and the engagement of key stakeholders. Therefore, to better understand the health system at the study site, a survey of the organizational capacity in the study area was conducted, along with interviews with the NCD counselors and the patients who visit the health facility. Following this, the selection of health care facilities was based on (1) the provision of NCD services, (2) the availability of health care staff such as NCD counselors and nurses to identify the patient participants for the study (by administering the Patient Health Questionnaire [PHQ-2]) [22], a screening tool to identify those who need further assessment for depression), (3) the engagement and support of senior managers, and (4) a sufficient number of patients with NCD attending the facility. Facilities were part of government-supported health services and were within a reasonable travel distance of the research team’s base to make the study feasible.

Since most NCD services are limited to providing care for cardiovascular and chronic lung diseases and diabetes (excluding cancer), the study focused on these disorders. Finally, a total of 6 NCD clinics were chosen for data collection.

### Eligibility Criteria for Trial Participants

The eligibility criteria for this study are given in Textbox 1.

**Inclusion and exclusion criteria for the study.**

**Inclusion criteria**

- Aged 18 years or older.
- Diagnosed with cardiovascular disease, chronic respiratory disease, or diabetes (type 1 or 2).
- Current diagnosis of depression (confirmed with a Patient Health Questionnaire [PHQ-9] [23] score of ≥10).
- Willing to participate and able to attend therapy sessions in person or by telephone.
- Other mental or physical illness comorbidities will not be a reason to exclude unless the patient is judged to be too unwell to participate.

**Exclusion criteria**

- Already receiving psychotherapy for depression. Being on antidepressant medication will not, however, be a reason to exclude.
- Lacking the capacity to provide informed consent.
- Unable to take part in therapy because of cognitive impairment or the severity of a mental or physical illness.

### Intervention Arm

Participants in the intervention arm will receive the BIP, delivered by trained nonmental health specialists (BA counselors) who will be specifically recruited for the study. BA counselors will be supervised by a mental health specialist.

The BIP will be delivered over 6 sessions lasting 30-40 minutes over a period of 6-12 weeks, with a minimum of one week between sessions. All sessions will be delivered remotely, by telephone. An alternate telephone number will be collected at the time of recruitment to ensure the unhindered delivery of BA sessions to the client.

The components of BIP include structured sessions where participants, with the BA counselors, plan and identify 3 types of activities. The first one includes routine and necessary activities that are done either regularly or on a daily basis, for example, getting fresh, taking a bath, cooking, doing household chores, etc. Second, social activities involve interacting with other people, for example, meeting or chatting with people from the neighborhood, etc. And third, pleasurable activities include activities that one enjoys and has fun with. For example, reading a newspaper or book, watching television (serials and movies), etc. The BA counselor and the participant will both have a flipbook for guidance and a better understanding of the activities during the session, as it contains a pictorial representation of each type of activity. The BA counselor will follow a manual and maintain the session logs of the participants. The BA counselor in the session log will not only maintain the session details but also keep a record of the daily activities done by the participants and the mood associated with doing these activities through telephonic follow-up in between the sessions. Another reason this is done is to check if the participants are doing the activities or if they are facing any challenges while doing them.

### Control Arm

Participants in the control arm will receive an EUC leaflet describing depression and its treatment and signposting, including providing contact details (addresses and telephone numbers) to help access usual care for depression. They will also be provided with 1-time brief advice on the same from the research assistants (RAs) remotely.

### Outcomes

The outcomes for the feasibility trial relate to (1) the acceptability and feasibility of recruitment and data collection and (2) the acceptability, feasibility, and implementation of the BIP.

Outcomes relating to the feasibility and acceptability of recruitment and data collection are (1) the rates of recruitment into the trial and retention at 3 months (RQ1), (2) the proportion of participants recruited who are randomized (RQ2), and (3) the completeness of data collection (percentage of planned measures completed) (RQ2) at:

- Baseline: demographic data, depression symptoms—PHQ-9 [23], negative emotional state of depression—depression subscale of Depression Anxiety and Stress Scale (DASS-21) [24], GAD-7 [25], health-related quality of life (EQ-5D-5L) [26], measures of potential mediators, for example, knowledge, intention, beliefs of 3 activities (routine and necessary, social, and pleasurable activities), and questions on how doing these activities will affect their NCDs, Premium Accredited Activation Scale (PAAS) [27], and adverse events.
- 3 months: depression symptoms—PHQ-9 [23], negative emotional state of depression—depression subscale of DASS-21, GAD-7 [25], health-related quality of life (Euroqol-EQ-5D-5L) [26], measures of potential mediators, for example, knowledge, intention, and beliefs of 3 activities (routine and necessary, social, and pleasurable activities), and questions on how doing these activities affected their NCDs, PAAS [27], and adverse events.

Outcomes relating to the acceptability, feasibility, and implementation of the BIP are described below in the process evaluation.

### Trial Sample Size

The sample size is set to enable reliable estimation of recruitment and retention rates and to adequately explore the feasibility of data collection and intervention delivery in this feasibility trial. A total of 56 participants will be recruited for this study. This should be adequate to estimate a participation rate of 20% and a follow-up rate of 80% to within a 95% CI (with SD of 5% and 10%, respectively).

### Trial Recruitment and Consent Procedures

The BEACON study flowchart can be found in Multimedia Appendix 2.

Participating sites were asked to implement depression screening by NCD health care staff, such as NCD counselors and nurses, using the PHQ-2 [22], as part of routine clinical practice. The PHQ-2 is a simple screening tool with minimal training and time requirements. The training was provided by the study team, and staff were asked to use the PHQ-2 with every patient attending the NCD service.

Staff (NCD counselors and nurses) will be asked to give information about the study and screen the patients for PHQ-2 [22] after obtaining their written consent. The participants who scored ≥3 will be qualified for the next level of screening. Those who indicated they were interested in participating in the feasibility trial were approached by RAs for the next level of screening by administering the PHQ-9 [23].

Recruitment of the willing participants by RAs was done remotely by telephone, mobile phone, internet, or video calling, as was convenient for the participant. Accordingly, verbal informed consent will be obtained before administering the PHQ-9, along with consent for participation in the feasibility trial. Those screening positive for depression on PHQ-9 (score ≥10) were invited to participate in the feasibility trial and provided detailed information about the purpose of the study and trial procedures either face-to-face or remotely. Furthermore, a suicide risk assessment was conducted at this point, where the participant was asked whether they have had thoughts of harming themselves or wished they were dead in the past week. If they say no, then the recruitment process will continue. Final verbal consent for participating in the trial will be obtained. The BEACON patient information sheet and consent forms can be found in Multimedia Appendix 3.

But if the answer is yes to the question of whether they have had thoughts of harming themselves or wished they were dead in the past week, then further questions on plans, actions, and prevention are to be asked, and based on the responses, the participants will be categorized into level A (mild), level B (moderate), and level C (severe). Three-week follow-ups by the RAs will be done, and then the decision on whether to recruit the patient or not will be made by the research team. The details about the safety considerations followed in the trial can be found in Multimedia Appendix 4.

Participants were free to withdraw consent and leave the trial at any time without giving a reason. They were able to withdraw by letting any member of the research team know that they wished to do so. If a participant withdrew consent to participate, no further data were collected from them. However, data collected up to the point of withdrawal were retained and used in the analysis, except where withdrawal of consent for the use of this data is explicit, in which case all data will be destroyed.

### Randomization

Once consent has been secured, eligible participants complete a baseline questionnaire and are then randomized (BIP or EUC) by the trial manager or research fellow who will be based at the central office. Participants will be allocated in a 1:1 ratio using simple randomization without stratification. Treatment allocation was concealed from the study team at the point of recruitment using an automated computer data entry system, administered remotely by York Trials Unit, and using a computer-generated randomization sequence generated by an independent statistician (using Stata; version 16 or later; StataCorp).

### Quantitative Data collection

Baseline and 3-month follow-up data were collected by RAs. The SPIRIT (Standard Protocol Items: Recommendations for Interventional Trials) schedule of enrollment, interventions, and assessments can be found in Multimedia Appendix 5. Quantitative data were collected with predesigned and tested data collection forms, using tablets to improve efficiency and minimize the risk of errors in data entry. Data will be anonymized, replacing identifiable personal data with unique study participant identification numbers, with the key only available to the local research team, principal investigator, and manager. Data will be secured and transferred safely, in line with the University of York data management policies and procedures. The completed SPIRIT checklist can be found in Multimedia Appendix 6.

### Quantitative Data Analysis

Analyses will be conducted using Stata software. The number of participants who were screened, randomized, treated, and followed up for the primary outcome (PHQ-9) [23] will be reported in a CONSORT (Consolidated Standards of Reporting Trials)-style flowchart [28]. The number of study completers, by trial arm, will be calculated as a proportion of the number randomized at baseline.

The proportion of baseline and outcome measures completed out of those planned and presented by the treatment allocation arm will also be calculated. The normality of the data will be assessed, and based on that, an independent *t* test or Mann-Whitney *U* test will be used to compare the intervention and the EUC arm.

### Process Evaluation

The 3 key functions of a process evaluation (mechanisms of impact, context, and implementation) will underpin our approach for the feasibility trial [29]. The qualitative inquiry will be used in combination with quantitative data to gain a more complete picture.

### Survey (Implementation and Mechanisms of Impact)

To capture the views and experiences of participants, all participants will complete a brief quantitative survey at a 3-month follow-up. Participants in the intervention arm will be asked about their engagement with and acceptability of BIP, views on remote delivery, as well as the perceived impact on their depression symptoms. Control arm participants will answer these questions in relation to the EUC leaflet. Participants in both trial arms will also complete some questions to assess the acceptability of trial processes.

Potential mediators of intervention impact will be measured at baseline and 3 months: intentions, beliefs about capabilities, and beliefs about outcomes. These have been selected to reflect the hypothesized mechanisms of action of BA [30].

### Qualitative Data Collection: Interviews (Implementation, Mechanisms of Impact, and Context)

A subsample of 16 participants will be interviewed. We will purposively sample to ensure a mix of participants (intervention and control arms, men and women, aged 60 years or younger and aged 60 years or older, and completed or dropped out of BIP). The interviews will seek in-depth feedback on the topics explored in the brief survey as well as explore individual and context barriers and drivers to performing the 3 types of activities (routine and necessary, social, and pleasurable).

Once delivery of the BIP is completed, a group interview will be conducted with the 4 BA counselors to explore their experiences, including the acceptability of the interventions and the barriers and drivers to delivery, including contextual factors such as remote delivery.

All interviews will be conducted in the local language, face-to-face using topic guides, and digitally audio-recorded. A hermeneutics approach, which encourages participants to discuss features of the intervention to elicit data on their experience and evaluation of its delivery or receipt, will be used with trial participants, family members, and health care staff [31].

Trial participants will provide written informed consent for these interviews within their trial consent.

### Fidelity Index (Implementation)

Fidelity in delivering BIP will be assessed using a fidelity index. This index consists of 2 subindices, namely, the adherence index, which assesses adherence to delivering the content of the 6 BIP sessions, and the interaction index, which assesses the level of counselor-participant with which the intervention was delivered. Both indices are scored on a 3-point Likert scale (0=not implemented, 1=partially implemented, and 2=fully implemented).

All BIP sessions will be audio-recorded. We will then perform the fidelity checks by reviewing the audio recording of a subsample (n=18) of sessions. These sessions will be purposefully selected to include a mix of sessions 1-6, delivered by all BA counselors early and late in the feasibility trial.

### Fidelity Data Analysis

The quantitative data from the fidelity index and questionnaire will be analyzed using descriptive statistics, including means and SD for continuous variables and absolute and relative frequencies for categorical variables.

### Qualitative Data Analysis

Interviews will be transcribed verbatim, translated into English, and analyzed using the Framework Approach [32], which is particularly useful for understanding and improving programs and policies and when multiple researchers are working with the data [33]. Excel (Microsoft Corp) software will aid in data handling.

Integration of interview findings with respective questionnaire data will be done using a “triangulation protocol” [34]. Methodological triangulation, where more than one research method or data collection technique is used [35], will be used in this study.

### Economic Evaluation

The feasibility of undertaking a full cost-effectiveness analysis will be assessed. This will primarily investigate the acceptability and resource use associated with capturing data for a cost-effectiveness evaluation in the full trial.

The team will capture costs associated with depression in NCD, such as delivery or receipt of the BIP. Costs will include the time to deliver the BIP and the cost of the materials used. And the costs of training the BA counselors to deliver the BIP will be calculated. Training requires the “staff time” of the trainer plus staff travel costs. Staff time is based on the salary of the trainer and allocated on a cost-per-minute basis, plus costs of materials.

Diaries to capture resource use, to be completed by patients and BA counselors, will be developed by the research team. Participants will be asked to complete these on a monthly basis for 3 months.

A pro forma to capture the costs of delivering the BIP incurred by the host organization will be completed by service managers, including staff salaries, time, and costs of estates and materials. An average cost per case per trial arm for treatment and the control group will be calculated.

Health service use will be assessed by asking pretested questions on contacts with doctors and nurses, hospital admissions, pharmacy visits, drug use, etc.

Quality of life will be assessed using a quality of life questionnaire (EQ-5D-5L) at baseline and 3 months. This information can be used as a quantitative measure of health outcomes as judged by the individual respondents. A higher score represents a better-perceived health state. The data will be checked for completeness.

### Data Management

The following data will be collected from feasibility trial participants (at baseline and 3-month follow-up): demographic data and validated instrument measurements of mental and physical health. The process evaluation of the feasibility trial will collect data from interviews with trial participants and BA counselors, a sample of audio-recorded BIP sessions, and drop-out data. Health economic data will be collected to provide evidence of the costs of providing the BIP in the host institution.

All study data will be stored in accordance with the General Data Protection Regulation and the University of York data management policies. Electronic data will be password-protected and stored on secure servers at the country’s partner institutions and the University of York.

Unique study identification (ID) numbers, allocated by the research team, will be used to anonymize data on electronic documents containing data from the trial and interviews. Paper-based documents, for example, consent forms and hard copies of transcripts, as well as any research participant personal data collected, will be stored in locked filing cabinets at the partner institutions.

Study documents (paper and electronic) at the research sites will be retained in a secure (locked when not in use) location during and after the study finishes. Interview recordings will be transferred from the digital recorder to a secure server as soon as possible after the interview has taken place. Once the transferred recording has been checked to ensure audibility, the original will be deleted from the digital recorder. All essential documents, including source documents (eg, transcripts), will be retained for a minimum period of 10 years after study completion, in accordance with the University of York Research Data Management Policy. This is deemed to be sufficient time for any queries arising from the findings to be answered, for example, queries arising from the publication of the findings. They will be retained in secure locations (locked when not in use) at the University of York and at the partner institutions.

The Department of Health Sciences at the University of York has a backup procedure approved by auditors for disaster recovery. There will be a separate archive of electronic data performed at the end of the study to safeguard the data and comply with regulatory requirements. The access, use, and storage of sensitive or confidential data will be conducted in accordance with the University of York Data Security Policy and Handling Sensitive Data Guidance. All sensitive or confidential data used for BEACON will be encrypted.

### Data Monitoring

Data will be monitored for quality and completeness by the delegated researchers at the study site, followed by a second check by researchers at the University of York using verification, validation and checking processes. Missing data will be pursued until the study’s end unless it causes any distress to the participant contacted. Data will be reported to the Health Ministry Screening Committee and Ethics Committee as required.

### Adverse Events

Working with a potentially vulnerable group of participants has many ethical implications. We have addressed them in our ethics application. The main adverse events anticipated in this study, both during the recruitment phase and during the delivery of the BIP, are self-harm and suicide risk. Therefore, standard operating procedures to address these adverse events will be adapted by the study team.

#### Informed Consent

As the recruitment is done at 2 levels, written consent will be obtained at first-level screening using PHQ-2 [22] by the staff (NCD counselors and nurses) and verbal consent for the next level screening using PHQ-9 [23] by the RAs.

#### Privacy and Confidentiality

Data will be anonymized, replacing identifiable personal data with unique study participant identification numbers, with the key only available to the local research team and the principal investigator.

### Community Engagement

A community advisory panel will provide community engagement and involvement for the duration of the study. This panel consists of patient and family representatives and community and voluntary sector advocates interested in improving the care of common mental disorders and NCDs. The panel has already given feedback on:

- The accessibility and feasibility for patients of the proposed BIP intervention, including remote delivery.
- Delivery of BA counseling by the NCD counselors.
- The acceptability of proposed methods for the feasibility trial.

The feedback and suggestions were incorporated into the intervention development and delivery plans of the study. The panel will continue to provide feedback, providing an important perspective on the delivery of the study and its findings.

### Dissemination

Feasibility study results will be disseminated by engaging end users of our research, including policy makers and commissioners, by holding knowledge transfer and dissemination events at key stages. Lay summaries and fact sheets of findings are distributed using social media, newspapers, and a project website, alongside publications in academic journals, to reach a range of audiences.

### Ethical Considerations

Ethics and other relevant approvals on the protocol have been secured from the University of York Health Sciences Research Governance Committee (HSRGC/2020/418/B; November 27, 2020); the Health Ministry Screening Committee, India (2020-9330; June 12, 2020); and the ethics committee, NIMHANS’s Behavioral Sciences Division (NIMHANS/EC [BEH. SC.DIV.] 22nd MEETING/2OI9; February 3, 2020). The trial is also registered under the Clinical Trial Registry of India (CTRI/2020/05/025048; May 6, 2020).

Approvals for necessary amendments adjusting to the COVID-19 pandemic situation have been provided by the institutional ethics committee, Behavioral Sciences Division, NIMHANS.

The following amendments are made to the protocol:

- In the first amendment to the protocol dated September 25, 2020, the focus group discussions and interviews as part of the survey of organizational capacity were shifted from face-to-face to telephonic mode.
- In the second amendment to the protocol dated May 15, 2021, the recruitment process was shifted from face-to-face to telephonic mode, and NCD counselors were involved in preliminary screening. A total of 4 BA counselors of the same cadre as the NCD counselors were recruited into the study to deliver the BA sessions.
- In the third amendment to the protocol dated January 27, 2022, process evaluation interviews are to be conducted at 8 weeks instead of 12 weeks, that is, immediately after the participants in the intervention group complete their 6 BA sessions and in the control group at 8 weeks after the participants receive their brief advice from the RAs postbaseline assessment.

Participants in the intervention group will receive INR 250 (US $3.01) per session, and participants in the EUC group will receive INR 250 (US $3.01) for attending the one-time brief advice.

## Results

The BIP was developed beginning on November 28, 2020, and it was finished on August 18, 2021. The quantitative data-gathering process began on August 23, 2021, and was finished on December 10, 2021, following the acquisition of approval from all pertinent agencies. Process evaluation (qualitative data) collection is ongoing. Both the qualitative and quantitative data analyses are ongoing.

## Discussion

### Overview

This research study overall aims to address the evidence gap regarding the use of BA for depression in people with NCDs by adapting BA interventions to the Indian context, testing the acceptability, feasibility, and implementation of adapted BA intervention, and testing the feasibility of a randomized controlled trial evaluation of BIP for the treatment of depression compared with EUC.

Effective treatment of depression in NCDs offers the potential to improve mental and physical outcomes for this population and, additionally, maximize the efficient use of health care resources. To bridge the treatment gap globally, psychological therapies must be scaled up, which necessitates their effective use in many cultural contexts. An increasing number of studies have examined the degree to which cultural adaptation enhances the acceptability and effectiveness of psychological therapies among populations from various cultural backgrounds. The data are still unclear at this stage because there are not enough studies that analyze the many types of changes involved in cultural adaptation [36]. If shown to be effective, the approach of integrating treatment for a mental disorder within NCD care will also provide the opportunity to harness physical health resources to reduce the large mental health treatment gap, reduce stigma for people with mental illness, and add to the data on cultural adaptation of interventions such as BA.

Several of the issues with conventional psychological therapy may be resolved through remote therapy. Because of their accessibility and simplicity, telephone-based therapies are a popular research topic in the field of therapeutic communication and may provide important advantages. Data suggests that a therapeutic connection and successful treatment outcomes may not require the colocation of a patient and therapist. Nonetheless, the evidence is scant in both amount and quality [37]. Therefore, this study might provide evidence of how well BA can be delivered remotely and whether this approach can be further scaled up.

If the feasibility trial demonstrates that the BIP is feasible and potentially beneficial, we will be able to make further adaptations to the BIP and test the intervention in a full trial.

### Limitations

Given the COVID-19 situation, BA counselors of the same cadre as the NCD counselors were recruited into the study to deliver the BA sessions, which can be a drawback as there is no sense of familiarity, which might affect intervention delivery.

### Future Directions

This work will support a future definitive trial evaluation of BA for depression in NCD in India.

### Conclusions

This study has the potential to provide evidence that would be useful to bridge the treatment gap for mental disorders, especially in resource-, infrastructure-, and system-constraint countries such as India. BEACON attempts to deliver BA for depression in NCDs through trained NCD (BA) counselors integrated within the NCD clinics to reduce this gap. The findings from this study will help understand the impact of the BA intervention for depression in NCD in countries such as India. Also, the BIP may serve as a culturally validated intervention package for the Indian population for future use.

## Appendix

Multimedia Appendix 1 Beacon feasibility trial Gantt chart.

Multimedia Appendix 2 Beacon study flow chart.

Multimedia Appendix 3 Beacon patient information sheet and consent forms.

Multimedia Appendix 4 Safety considerations.

Multimedia Appendix 5 SPIRIT (Standard Protocol Items: Recommendations for Interventional Trials) schedule of enrollment interventions and assessments.

Multimedia Appendix 6 SPIRIT (Standard Protocol Items: Recommendations for Interventional Trials) checklist.

## Abbreviations

BA: behavioral activation
BIP: BEACON intervention package
CONSORT: Consolidated Standards of Reporting Trials
DASS-21: Depression Anxiety and Stress Scale
EUC: enhanced usual care
GAD: generalized anxiety disorder
INDEPENDENT: Integrating Depression and Diabetes Treatment
LMICs: low- and middle-income countries
mhGAP: Mental Health Gap Action Program
NCD: noncommunicable disease
NIMHANS: National Institute of Mental Health and Neuro Sciences
NPCDCS: National Programme for Prevention and Control of Cancer, Diabetes, Cardiovascular Diseases, and Stroke
PAAS: Premium Accredited Activation Scale
PHQ-9: Patient Health Questionnaire-9
RA: research assistant
RQ: research question
SPIRIT: Standard Protocol Items: Recommendations for Interventional Trials
WHO: World Health Organization

## Data Availability

All data generated or analyzed during this study are included in this study and its Multimedia Appendices.
