# Supplementary figures and images for: Behavioral Activation for Comorbid Depression in People With Noncommunicable Disease in India: Protocol for a Randomized Controlled Feasibility Trial
Source: JMIR Res Protoc. 2023 Nov 16;12:e41127. doi: 10.2196/41127 (PMC10690525; doi:10.2196/41127)

## BEACON study flow chart:

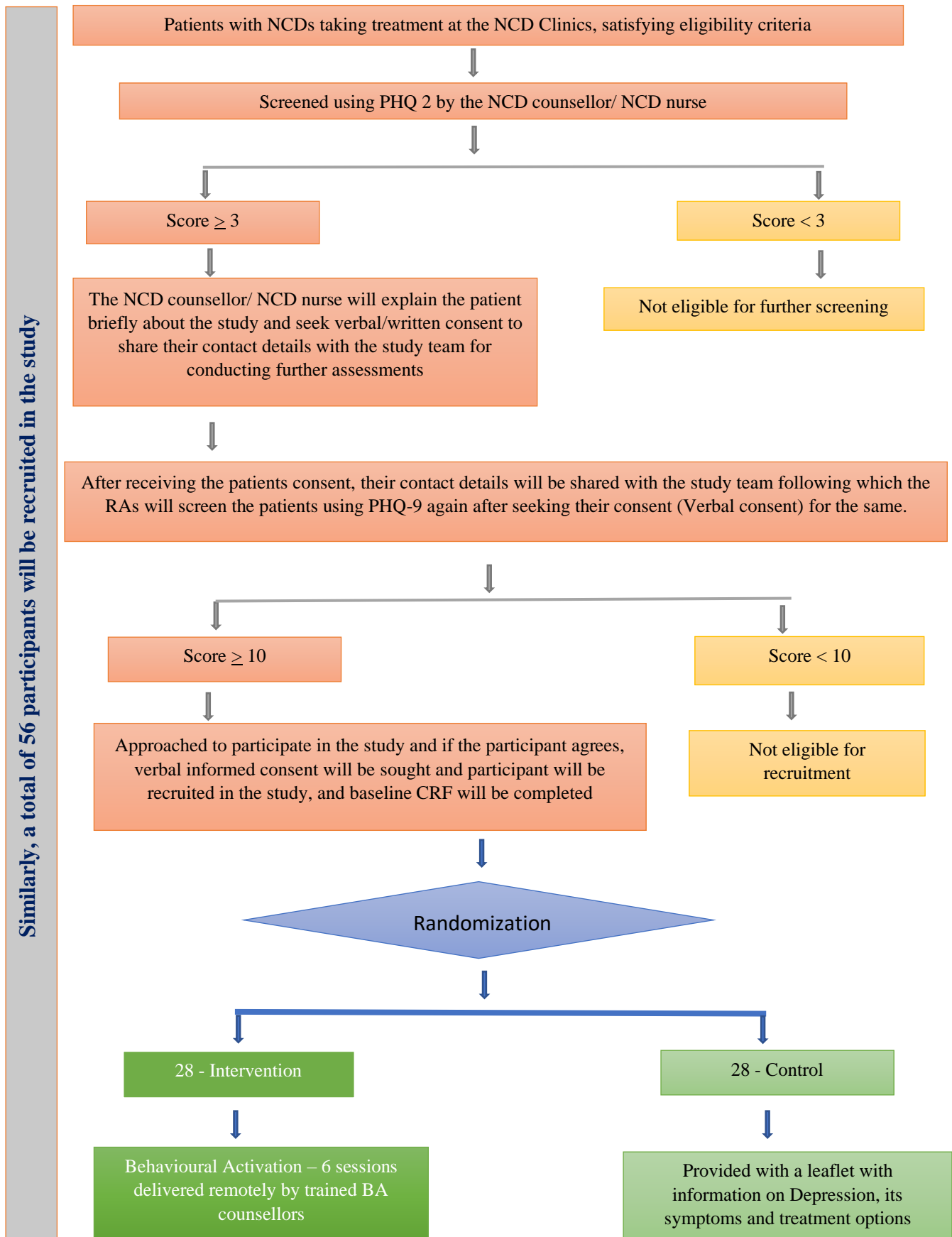

Supplement: Multimedia Appendix 2 [file resprot_v12i1e41127_app2.pdf]
