# Supplementary material for: Behavioral Activation for Comorbid Depression in People With Noncommunicable Disease in India: Protocol for a Randomized Controlled Feasibility Trial
Source: JMIR Res Protoc. 2023 Nov 16;12:e41127. doi: 10.2196/41127 (PMC10690525; doi:10.2196/41127)
Supplement: Multimedia Appendix 3 [file resprot_v12i1e41127_app3.docx]

**Patient Information Sheet – for screening using PHQ-2 at NCD clinic**

*Dear Participant,*

The BEACON study (Behavioural Activation for Co-morbid Depression in Non-communicable diseases) is sponsored by the University of York, UK, and funded by the National Institute of Health Research (NIHR) in the UK. Dr Arun Kandasamy, Additional Professor, Department of Psychiatry, NIMHANS, is the Principal Investigator of the project.

**Purpose of the study:**

Many people live with long-term physical illnesses (also called non-communicable diseases - NCDs) such as diabetes, obstructive respiratory conditions, heart-related diseases and cancers. People with NCDs may also develop mental health problems from time to time such as depression. However, at the moment the NCDs care services in government health facilities do not offer treatment for depression.

This research is an attempt to assess if simple behavioural therapy for depression could be introduced within NCD care at this health facility. It is to see if people find this acceptable and what changes are needed to make it suitable for people who have long-term health problems.

**Screening using PHQ-2:**

As the first level of screening, we will be asking you only two questions from the Patient Health Questionnaire (PHQ-2). If you are willing to undergo this screening, please provide your consent below.

**Consent form:**

Study Title: Behavioural Activation for Co-morbid Depression in Non communicable diseases (BEACON) Study.

Principal Investigator: Dr Arun Kandasamy, Additional Professor of Psychiatry, NIMHANS, Bangalore 560029

I have read/ been read the Project brief/ Information sheet by the NCD Counsellor/ NCD nurse and I have understood the objectives of this study. I have had the opportunity to ask questions about it and any questions I have asked, have been answered to my satisfaction.

I understand that my participation is voluntary and I consent voluntarily to undergo the first level of screening using PHQ-2 and I also understand that I have the right to withdraw from the study at any time without it affecting me in any way.

I understand that my involvement is strictly confidential and all the information collected as a part of the study will be kept strictly confidential.

I authorize that the information provided by me can only be looked at by authorized persons involved in this study. I also understand that if I have any concerns regarding this study, I can contact the Principal Investigator.

**Details of the Participant:**

Name:

Address:

Signature/ Thumb impression of the study participant:

Date:

Contact Number:

**Details of the person obtaining consent:**

Name:

Designation:

Signature:

Date:

Place:

**Participant Information Sheet for sharing their contact details with the study team for second-level screening using PHQ-9:**

Dear Participant,

You have been tested positive for PHQ-2 and require further evaluation using PHQ-9. If you share your contact details with the BEACON study team, the Research Assistants will contact you and screen you for PHQ-9. And if you screen positive for PHQ-9, then the Research Assistants will seek your verbal consent and recruit you into the study. By offering you the BA support and asking you to speak with a researcher about your health conditions and the BA support, we hope to understand more about what might help this group of people. If you are willing to get the assessments done, then please provide your consent below.

**Consent form:**

Study Title: Behavioural Activation for Co-morbid Depression in Non communicable diseases (BEACON) Study.

Principal Investigator: Dr Arun Kandasamy, Additional Professor of Psychiatry, NIMHANS, Bangalore 560029

I have read/ been read the Project brief/ Information sheet by the NCD Counsellor/ NCD nurse and I have understood the objectives of this study. I have had the opportunity to ask questions about it and any questions I have asked, have been answered to my satisfaction.

I understand that my participation is voluntary and I consent voluntarily to share my contact details with the study team for conducting further assessments and I also understand that I have the right to withdraw from the study at any time without it affecting me in any way.

I understand that my involvement is strictly confidential and all the information collected as a part of the study will be kept strictly confidential.

I authorize that the information provided by me can only be looked at by authorized persons involved in this study. I also understand that if I have any concerns regarding this study, I can contact the Principal Investigator.

**Details of the Participant:**

Name:

Address:

Signature/ Thumb impression of the study participant:

Date:

Contact Number:

**Details of the person obtaining consent:**

Name:

Designation:

Signature:

Date:

Place:

**Patient Information Sheet**

**BEACON study: Feasibility Randomized Controlled Trial of Behavioural Activation for Comorbid Depression in Non-Communicable Diseases**

#### **Information Sheet for Beacon Feasibility Trial Participants (Phase II)**

**Background and rationale for the study:**

The rising burden of depression and non-communicable disease multimorbidity is an increasing global challenge, largely neglected by healthcare services, particularly in low- and middle-income countries (LMIC). Co-existence of depression and chronic physical disease is common and worsens outcomes for both the mental and physical disorder. Depression is amenable to treatment; effective interventions include a range of pharmacological and psychological therapies. Encouragingly for resource-constrained health systems, brief therapies, such as behavioural activation, have been shown to be as effective as more resource-intensive therapies for the treatment of depression in the general population. Several clinical guidelines recommend use of these brief therapies, including for depression in people with physical health conditions. However, their feasibility and effectiveness for depression in patients with non-communicable diseases is not known. Phase II of the study is to test acceptability, feasibility and implementation of BEACON in a feasibility randomised controlled trial.

**Who is organizing and funding the research?**

The study is sponsored by the University of York, UK, and funded by the National Institute of Health Research (NIHR) in the UK. The study is a collaboration of researchers at the University of York and the National Institute of Mental Health and Neurosciences (NIMHANS), Bangalore, India.

The research team is led by Dr Najma Siddiqi (Phase II), Department of Health Sciences, University of York, UK, Jerome Wright (Phase I), Department of Health Sciences, University

of York, Dr. Arun Kandasamy and Prof Pratima Murthy, National Institute of Mental Health and Neurosciences (NIMHANS), India.

**What is the purpose?**

Many people live with long-term physical illnesses (also called non-communicable diseases -NCDs) such as, diabetes, obstructive respiratory conditions, heart related diseases and cancers. People with NCDs may also develop mental health problems from time to time such as depression. However at the moment the NCDs care services in government health facilities do not offer treatment for depression.

We are running a research to assess if simple behavioural therapy for depression could be introduced within NCD care at this health facility. We have previously designed a form of counselling called Behavioural Activation (BA support) to help people with long-term health problems to maintain a positive mood. We now want to see if people find this acceptable and what changes we need to make to this to make it suitable for people who have long-term health problems. By offering you the BA support and by asking you to speak with a researcher about your health conditions and the BA support, we hope to understand more about what might help this group of people.

**Why have you been chosen?**

We are approaching adults (≥18 years old) diagnosed with cardiovascular disease, chronic respiratory disease or diabetes (type 1 or 2) and with and without a diagnosis of depression (confirmed with RA administered PHQ-9 score of ≥ 10) attending NCD services at the selected facilities, resident in the same district.

**What is the estimated duration of research participation?**

We anticipate that BEACON will be remotely delivered over six sessions, 30-40 minute time duration, over a period of 6 to 12 weeks, with a minimum of one week between sessions. Sessions could potentially be offered by BA Counsellor in the treatment facility, depending on findings during the development work.

**What will be involved if you take part?**

If you are interested to take part in the study, your suitability for the study will be assessed by the researcher by asking you questions about how you felt in the last two-weeks. The process will take maximum of ten minutes and will be done remotely. If you are suitable to take part and you are happy to proceed with the study you will be randomly assigned to receive either only enhanced usual care for depression or BA support along with it. The randomisation sequence will be generated by a statistician at the York Trials Unit using Stata software. Participants will be randomised on a 1:1 basis to intervention and control arms by investigators from NIMHANS.

In both cases, BA Counsellors would be working with you to improve your physical and emotional health and wellbeing. The BA counsellor will contact you to arrange a convenient time to meet you or speak with you. The meetings will take place over the telephone. You will speak with the BA Counsellor up to a total of six times, normally on a weekly basis, within the period of three months. Each time you speak to the BA Counsellor, it will last about 30-40 minutes.

Under Enhanced Usual Care you will receive a leaflet describing depression and its treatment and contact details of providers for accessing care for depression. While under BA support, you will receive enhanced usual care plus the BA support as refined and adapted through BEACON Study Phase-1 co-design and refinement activities.

Once you agree to participate in the study, the RA will ask you to provide the verbal consent which will be audio/video recorded. The RAs will read the consent form to you and collect your personal details. After the receipt of the verbal consent, a detailed assessment with regard to your physical and mental health will be conducted by the RA using:

1. Standard questionnaire for depression and anxiety symptoms.
2. Standard questionnaire for your physical health and functioning.

At 3 month after your last meeting session with the BA Counsellor, you will be invited to take- 1. Standard questionnaire based assessments for depression and anxiety symptoms

1. Standard questionnaire based assessments on your physical health and functioning
2. Assessments to record any event within 3 months that might have had adverse impact on you.

We may ask for your permission to audio record some of the meeting sessions you have with health-worker. Members of the research team will then listen to these recordings to help them further develop the support. If you do not wish these sessions to be recorded you can still take part in the study.

You may be asked to consent for a face-to-face or telephone interview at 3 months after your first meeting with the health-worker. The interview will assess your experience and satisfaction with study participation and sharing of information. If you are assigned to BA support group, you will be asked to give your opinion about if you found the BA support acceptable and feasible to be offered at the NCD facility. Besides, you will be asked to share your opinion on how the BA support should be offered.

The interview shall also inquire about your earlier utilization of healthcare services for NCD condition and depression including contacts with doctors/nurses, hospital admissions, pharmacy visits and drug use and physiotherapy. With your permission, the interview will be

audio-recorded but you will not be identifiable from these recordings at any later usage of this information. If suicidality or any other significant mental health problems identified during the course of the study, the subjects will be referred to the nearest mental health facility for appropriate care

**What are the potential risks or discomforts from participating in the study?**

No risks or discomforts involved during participation in the study

**What are the benefits of participating in the study?**

You will have the opportunity to talk about your experience of managing a NCD, and to share your ideas on how treatment services can be improved, including the introduction of simple

behavioural therapy. Some people find talking helpful. We will reimburse your travel costs. Your decision to participate or not in the study will not influence the treatment of NCD which you are receiving currently. In case of any health-related issues being identified during the interviews, you will be referred to the appropriate health care agency.

**Confidentiality**

If you decide to participate in the study, the trained non specialist counsellor will be delivering the intervention. No identifying information will be stored with your study data. Any paper copies (e.g. informed consent forms) will be stored in locked cabinets in locked offices at NIMHANS.

You will find that some of the questions that you will be asked when completing questionnaires are about your age, gender, marital status and occupation. This data will only be used to produce summaries of the characteristics of the people who have participated in the study. These summaries will not contain your name or any information that can be used to identify you.

You will be asked to write your name and sign at the bottom of an informed consent form.

However, the informed consent form and any other documents that we might have that contain any information that can be used to identify you will be stored securely, separated from the

study data. All data collected will be kept confidential in line with respective countries’ the Data Protection Act (India and UK).

Access to participants’ personal details will be restricted to research staff only. Monitors and auditors may also need to access the data. For example, the Indian Council of Medical Research may wish to monitor the study, in which case they will be granted access to private information

that identifies your name. At the end of the study, data will be securely archived at NIMHANS or the University of York, as appropriate for 10 years.

**What are the alternatives to participating in the study?**

Participation in the study is not mandatory and you can choose not to participate in the study without any consequence. If you do not wish to participate in the study but still want to get help for NCD or depression please talk to your treating health team.

**What will the study cost me and do I receive any compensation for participation in the study?**

Participating in the study is not expected to cost you anything, except taking time out of your normal schedule to complete the questionnaire and intervention sessions. For study visit or BACON study session, we will reimburse your actual travel expenses and also give you INR 250 as compensation for your time

**What if I have questions about the study?**

If you want to know more about the study or if you have a concern about any aspect of this study you may ask the researcher now. You can also contact the Principal Investigator of this study later, Dr Arun Kandasamy with any questions you might have either personally or by telephone at:

Dr Arun Kandasamy

Additional Professor

Centre for Addiction Medicine

Department of Psychiatry

National Institute of Mental Health and Neurosciences

Bangalore 560029, INDIA

Telephone number: +9108026995856

Email: [arunk@nimhans.ac.in](mailto:arunk@nimhans.ac.in)

**What if I have questions about participants’ rights?**

If you have any questions about your rights as a research participant, wish to complain, or have any concerns about how the study is being carried out, or any other aspects of your care during your participation in the study, you may contact the following

The Dean and Member Secretary,

Ethics Committee (Behavioral Sciences Division),

NIMHANS, Bangalore, India - 560029

Off: +9108026995004,

Email: [icmrhqds@sansad.nic.in](mailto:icmrhqds@sansad.nic.in)

**Do I have to participate in this study?**

You do not have to take part in the study if you do not wish to. Participating in this study is entirely voluntary. If you do not want to take part, your rights and the care that you are already receiving from your mental health care team will not be affected in any way. Even if you agree to participate in this study, you can still change your mind and decide not to participate at any time- you do not need to give any reason. If you decide to do so, you can inform the researchers. In that case, no further data will be collected from you. However, we will keep the data that we have already collected up to that point and use it for analysis, unless you clearly let us know that you do not wish for these data to be kept and used in this way, in which case all data will be destroyed

**What will happen to the results of the study?**

The results from this study will be presented as a report and also published in journals. We may also present the findings at conferences. However, you will not be identified in any reports, publications or presentations. If you wish to get feedback on findings and progress of the study please contact the country lead on the contact details provided above and we will give you this information. Any new information that affects the study or data that has clinical relevance to you (including incidental findings) will be made available to you. We will also inform your health care providers if you give us consent to do so.

**Ethical approval:**

The study has been approved by the Health Ministry Screening Committee, Government of India; Ethics Committee (Behavioural Sciences Division), NIMHANS, Bangalore India; and

the Health Sciences Research Governance Committee at the University of York in the UK. This study is also registered with the Clinical Trials Registry of India.

**Undertaking by the investigators**

Your consent to participate in the above study is sought. You have the right to refuse consent or withdraw the same during any part of the study without giving any reason. In such event, you will still receive best possible treatment, without any prejudice. We undertake to maintain confidentiality regarding the information obtained from the subject during the course of study. If you have any doubts about the study, please feel free to clarify with

- Dr. Arun Kandasamy, Department of Psychiatry, NIMHANS, Hosur Road, Bangalore –560029; 080-26995856/5250; Email: [arunk@nimhans.ac.in](mailto:arunk@nimhans.ac.in)
- Prof. Pratima Murthy, Department of Psychiatry, NIMHANS, Hosur Road, Bangalore – 560029; Off: 080-26995274/5998/5241; Email: pratimamurthy@gmail.com
- Dr. Gitanjali N, Associate Professor, Department of Clinical Psychology, NIMHANS, Hosur Road, Bangalore – 560029 ; Email ID: [gitanjali_n@yahoo.com](mailto:gitanjali_n@yahoo.com)
- Sr. Prof. Santosh Kumar Chaturvedi, Department of Psychiatry, NIMHANS, Hosur Road, Bangalore – 560029; 080-26995264/5250; Email: [skchatur@gmail.com](mailto:skchatur@gmail.com)
- Dr. Krishna Prasad M, Department of Psychiatry, NIMHANS, Hosur Road, Bangalore–560029; Off:080-26995864/5250; Email: [krishnadoc2004@gmail.com](mailto:krishnadoc2004@gmail.com)
- Prof. Dr. Nagaraja Rao Girish, Department of Epidemiology, Centre For Public Health, NIMHANS, Hosur Road, Bangalore–560029; Off:080-26995299; Email: [girishnrao@gmail.com](mailto:girishnrao@gmail.com)

**BEACON study: Behavioural Activation for Comorbid Depression in Non-Communicable Diseases – Phase II**

**Consent Form for remote recruitment of participants**

**Please add a tick (**✔**) to the boxes below when agreed by the participant:**

|  | A researcher verbally explained the background, purpose and procedures involved in this study. He/she provided me the webpage address where I can find all the relevant information of the study (*We might consider giving them address of IMPACT website).*  I have had an opportunity to ask questions or discuss any concerns about the study and participating in it with the researcher. | | | |
| --- | --- | --- | --- | --- |
|  | I understand that based on random chances for allocation to either trial group- I may only receive enhanced usual care for depression or this in combination with BA support for depression  I understand that I will have 30-40 minute long weekly meeting sessions with health workers for about six times over three months.  I understand that twice I will require to take certain physical and mental assessments during the study period and these assessments will be performed by the health-worker or researchers of the study | | | |
|  | I am aware that participating in this study is completely voluntary, and withdrawing from the study is always an option and does not require giving reasons for withdrawal or informing beforehand.  I am aware that not taking part in the study or withdrawing from it will not affect my current or future health care. | | | |
|  | I am also aware that I am free to decline answering any question that I may not wish to answer  I am aware that any information I provide, including personal data, will be kept confidential and stored securely.  I am aware that that the data collected during the study will be looked at by the research team (investigators and researchers involved in the data collection and analysis). It may also be looked at by representatives from regulatory authorities if they wish to monitor the study.  I am aware that my name will not be linked with the research materials, and I will not be identified or identifiable in any reports or research articles that result from the research.  I am aware that if I withdraw from the study, the information I have already provided will still be used for the research unless I specify that it should not be used in this way.  I am aware that some of the sessions might be audio recorded for the purpose of improving the quality of the BEACON intervention.  I am aware that I might be contacted after 3 months (1 month) from the date of the last session and some questions might be asked regarding my experience and satisfaction with different components of the study and the BEACON intervention | | | |
|  | I hereby confirm my voluntary participation in the BEACON study. | | | |
|  | I give permission to the research team and other monitoring agencies to have access to my records. | | | |
| Yes  No | I give researchers from the BEACON team permission to record my verbal consent, and  some of the sessions and interviews at 3 months follow up. | | | |
| Yes  No | I give researchers from the BEACON team permission to contact me again for  Follow up Questions. | | | |
| **Participant’s Name** | | **Yes/ No**  **Agreed to participate?** | | _ _ / _ _ /_ _ _ _  **Date** |
| **Researcher’s Name** | | **Researcher’s signature** | | _ _ / _ _ /_ _ _ _  **Date (dd/mm/yyyy)** |
|  | |  | |  |
|  | |  | | |
| **Patient Identification Number** | | |  |  |
