# Supplementary material for: Behavioral Activation for Comorbid Depression in People With Noncommunicable Disease in India: Protocol for a Randomized Controlled Feasibility Trial
Source: JMIR Res Protoc. 2023 Nov 16;12:e41127. doi: 10.2196/41127 (PMC10690525; doi:10.2196/41127)
Supplement: Multimedia Appendix 4 [file resprot_v12i1e41127_app4.pdf]

Safety Considerations

Research Procedures:

The researcher will provide assurance on participants anonymity, confidentiality and rights on refusing to answer any uncomfortable question/s, asking for a temporary break or stopping the interview altogether and / withdrawal from the study without any consequence. Even though least, but there is a possibility that a few questions might cause distress to the participant. In such a case, the researcher will seek help from an in-house mental health expert (Principal Investigator, PI) as per the need.

Adverse events:

Standard operating procedures (SOPs) on suicidality adapted for the study would be followed to assess and handle the levels of suicide risk (Level A – Mild, Level B – Moderate and Level C – Severe) of threats to participants own life as expressed through their thoughts, feelings or other signs.

1. **For recruiting participants with mild and moderate suicide risk into BEACON study:**

Risk assessment during recruitment:

Assessing the risk of self- harm/suicide:

- During recruitment, you must check for risk by asking participants the following question:
  "Have you had thoughts of harming yourself or wished that you were dead in the past one week?"

- If the participant indicates that they have NOT had such thoughts, then you should continue with the recruitment.

- If the participant indicates that they HAVE had such thoughts, then you must enact the Risk Protocol below.

Questions to ask & protocol if the risk has been identified:

- If the risk of self-harm/suicide is identified, advise the participant:
  "I see that you've said / you mentioned that…...... These are thoughts/feelings that people can have from time to time, but it's important to make sure you are receiving the right kind of support. So, if it's OK, I would now like to ask you some more questions that will explore these feelings in a little more depth."

- Ask the participant the six Exploring Risk Questions below.

- Make sure you document **verbatim** the participant's responses to the probing question **<u>and</u>** each of the six exploring risk questions to aid in establishing the level of risk.

**Details of disclosed thoughts (please record verbatim as far as possible)**<br><br><br>**Now ask the following six questions**	
**Plans**	
1. Do you know how you would harm yourself or try to end your life?<br>If **Yes** – details	Yes / No
2. Have you made any actual plans to harm yourself or end your life?<br>If **Yes** – details	Yes / No
**Actions**	
3. Have you made any actual preparations to harm yourself or end your life?<br>If **Yes** – details	Yes / No
4. Have you ever attempted to end your life in the past?<br>If **Yes** – details	Yes / No
**Prevention**	
5. Is there anything stopping you harming yourself or attempting to end your life at the moment?<br>If **Yes** – details	Yes / No
6. Do you feel that there is any immediate danger that you would act on these ideas about harming yourself or ending your life?<br>If **Yes** – details	Yes / No

During recruitment, the RAs must check if the patients had any suicidal thoughts in the past one week

No → Go to the next steps of the session

Yes ↓

Ask 6 exploring Questions and decide the level of risk

**Yes: Q5**<br>**No: All others**<br>**Level A**	**Yes: any Q 1-5**<br>**No: Q 6**<br>**Level B**	**Yes: Q6**<br>**No: Q5**<br>**Level C**

Level A – Action to be taken

- Explain the participant that it seems from the interaction that he/ she doesn't seem to act on the negative thoughts.
- Advise the participant to see the doctor.
- Inform the study team.
- **Three weeks of follow up needs to be done by the RAs (Check suicidality status)**
- **Participant can be recruited into the study if there is no risk of suicidality reported.**

Level B – Action to be taken

- Explain that they need to see the doctor.
- Take consent from the participant and inform the doctor.
- If the participant is not comfortable with the RAs informing the doctor, then encourage the participant to consult the doctor personally.
- Contact the study team
- **Three weeks of follow up needs to be done by the RAs (Check suicidality status).**
- **During the follow up if suicidal risk is not present, then consider recruiting the participant (recruitment shall take place on case-based analysis).**

Level C – Action to be taken

- Actively suicidal.
- Contact the study team and doctor immediately even if the participant doesn't consent to this.
- **Three weeks of follow up needs to be done by the RAs (Check suicidality status).**
- **Participant cannot be recruited into the study**

2. **For participants in the intervention arm: Risk assessment during each session:**

Assessing the risk of self- harm/suicide:

- During the session, you must check for risk by asking participants the following question:
  "Have you had thoughts of harming yourself or wished that you were dead in the past one week?"

- If the participant indicates that they have NOT had such thoughts, then you should continue with the session

- If the participant indicates that they HAVE had such thoughts, then you must enact the Risk Protocol below.

Questions to ask & protocol if the risk has been identified:

- If the risk of self-harm/suicide is identified, advise the participant:
  "I see that you've said / you mentioned that……… These are thoughts/feelings that people can have from time to time, but it's important to make sure you are receiving the right kind of support. So, if it's OK, I would now like to ask you some more questions that will explore these feelings in a little more depth."

- Ask the participant the six Exploring Risk Questions below.

- Make sure you document **verbatim** the participant's responses to the probing question **and** each of the six exploring risk questions to aid in establishing the level of risk.

**Details of disclosed thoughts (please record verbatim as far as possible)**<br><br><br>**Now ask the following six questions**	
**Plans**	
7. Do you know how you would harm yourself or try to end your life?<br>If **Yes** – details	Yes / No
8. Have you made any actual plans to harm yourself or end your life?<br>If **Yes** – details	Yes / No
**Actions**	
9. Have you made any actual preparations to harm yourself or end your life?<br>If **Yes** – details	Yes / No
10. Have you ever attempted to end your life in the past?<br>If **Yes** – details	Yes / No
**Prevention**	
11. Is there anything stopping you harming yourself or attempting to end your life at the moment?<br>If **Yes** – details	Yes / No
12. Do you feel that there is any immediate danger that you would act on these ideas about harming yourself or ending your life?<br>If **Yes** – details	Yes / No

During each session the BA counsellors must check if the patients had any suicidal thoughts in the past one week

No → Go to the next steps of the session

Yes ↓

Ask 6 exploring Questions and decide the level of risk

**Yes: Q5**<br>**No: All others**<br>**Level A**	**Yes: any Q 1-5**<br>**No: Q 6**<br>**Level B**	**Yes: Q6**<br>**No: Q5**<br>**Level C**

Level A – Action to be taken

- Explain the participant that it seems from the interaction that he/ she doesn't seem to act on the negative thoughts.
- Advise the participant to see the doctor.
- Inform the study team.
- Inform the respective NCD counsellor.
- **The RA's to conduct clinical assessment immediately and discuss with the PI about the about the same.**
- **PI, will make the decision whether to continue the session or not.**
- **As usual three follow ups will be done and regular updates will be given to the PI.**

Level B – Action to be taken

- Explain that they need to see the doctor.
- Take consent from the participant and inform the doctor.
- If the participant is not comfortable with the RAs informing the doctor, then encourage the participant to consult the doctor personally.
- Contact the study team
- Inform the respective NCD counsellor.
- **The RA's to conduct clinical assessment immediately and discuss with the PI about the about the same.**
- **PI, will make the decision whether to continue the session or not.**
- **As usual three follow ups will be done and regular updates will be given to the PI.**

Level C – Action to be taken

- Actively suicidal.
- Contact the study team and doctor immediately even if the participant doesn't consent to this.
- Sessions will be stopped.
- Inform the respective NCD counsellor.
- Inform the respective ASHA.
- Regular follow up to be continued.
