# Supplementary material for: Behavioral Activation for Comorbid Depression in People With Noncommunicable Disease in India: Protocol for a Randomized Controlled Feasibility Trial
Source: JMIR Res Protoc. 2023 Nov 16;12:e41127. doi: 10.2196/41127 (PMC10690525; doi:10.2196/41127)
Supplement: Multimedia Appendix 5 [file resprot_v12i1e41127_app5.docx]

**SPIRIT schedule of enrolment, interventions and assessments:**

|  | **STUDY PERIOD** | | | | | | | |
| --- | --- | --- | --- | --- | --- | --- | --- | --- |
|  | **Enrolment** | **Post-allocation** | | | | | | |
| **Time point (Weeks)** | -4 | 0 (Baseline) | 2 | 4 | 6 | 8 | 10 | 12 weeks after baseline |
| **Research/Treatment Appointment** | Research-only appt. | Session 1 | Session 2 | Session 3 | Session 4 | Session 5 | Session 6 | Research-only appt. |
| Delivery/data collection method | Face-to-face | Face-to-face or telephone | Face-to-face or telephone | Face-to-face or telephone | Face-to-face or telephone | Face-to-face or telephone | Face-to-face or telephone | Home visit or telephone |
| **ENROLMENT:** |  |  |  |  |  |  |  |  |
| Eligibility screen | √ |  |  |  |  |  |  |  |
| Informed consent | √ |  |  |  |  |  |  |  |
| Allocation | √ |  |  |  |  |  |  |  |
| **INTERVENTIONS:** |  |  |  |  |  |  |  |  |
| Intervention (BEACON + enhanced usual care) |  | **x** | **x** | **x** | **x** | **x** | **x** |  |
| Control (enhanced usual care) |  | **x** | **x** | **x** | **x** | **x** | **x** |  |
| **ASSESSMENTS:** |  |  |  |  |  |  |  |  |
| PHQ-9, DASS | √ | √ |  |  |  |  |  | √ |
| GAD-7, EQ-5D-5L, PAAS |  | √ |  |  |  |  |  | √ |
| Health economics data collection |  |  |  | √ |  | √ |  | √ |
| Adverse events |  |  |  |  |  |  |  | √ |
| Planned, completed and missed therapy appointments |  | √ | √ | √ | √ | √ | √ |  |
| Retention in treatment |  | √ | √ | √ | √ | √ | √ |  |
| Interviews |  |  |  |  |  |  |  | √ |
| Audio-recording of treatment sessions (randomly selected) |  | √ | √ | √ | √ | √ | √ |  |
